# Emotional indicators in young patients with Idiopathic Scoliosis: a study through the drawing of Human Figure

**DOI:** 10.1186/s13013-014-0024-5

**Published:** 2014-12-12

**Authors:** Elisabetta D’Agata, Manuel Rigo, Carles Pérez-Testor, Núria Casanovas Puigví, Carmina Castellano-Tejedor

**Affiliations:** Vall d’Hebron Research Institute, Passeig Vall d’Hebron, 119-129, 08035 Barcelona, Spain; Institut Elena Salvá, Vía Augusta 185, 08021 Barcelona, Spain; Universitat Ramon Llull, FPCEE Blanquerna and IUSM Vidal i Barraquer, C/Cister, 34, 08022 Barcelona, Spain; Independent Researcher, Barcelona, Spain

## Abstract

**Background:**

Investigating Health Related Quality of Life (HRQL) is considered determinant in patients with Adolescent Idiopathic Scoliosis (AIS) in clinical as in research field. The aim of the present study is to explore the most relevant aspects of personality of the patients with AIS and its relationship with HRQL.

**Method:**

50 patients (mean age = 16 years) were given a socio-demographic data questionnaire, the Human Figure Drawing (HFD) and SRS (Scoliosis Research Society) -22.

**Results:**

In Subtotal SRS-22, patients presented a mean value of 3.9. In HFD, half of these patients presented physical and/or emotional tensions with reference to the shoulders and almost all of them did not show any expression of aggressiveness. No relationship between personality and HRQL was confirmed. The older the patients were, the more body tension was discovered as well as the more concerns about their bodies they showed to have. There was also a correlation between growing old and a decreasing in Mental Health. Previous conservative treatment did not show any impact on personality or on HRQL.

**Conclusions:**

Patients with AIS suffer stress and general concern more frequently with the increase of age. We suggest an appropriate supportive treatment for this type of patients.

## Background

Adolescent Idiopathic Scoliosis (AIS) is a complex process of a three-dimensional spine and trunk deformity, which appears in otherwise healthy subjects, and can progress in relation to multiple factors, during any period of rapid growth [1].

Most of the studies about AIS have focused on investigating the Health Related Quality of Life (HRQL), due to the delicate age of development of scoliosis in these young patients, with the aim of assessing the impact of the deformity and of the treatments in their lives.

For this reason, specific instruments for patients with AIS have been created [2], as SRS (Scoliosis Research Society) -22 [3] and QLSD (Quality of Life in Spinal deformities) [4]. Among all the instruments, the SRS-22 is the most widely used and translated into many languages.

However, in AIS the studies interested in investigating the personality traits of patients with scoliosis are limited. To know the personality of these patients may be useful to identify changes over treatment, modify the intensity of brace treatment, indicate a supportive psychological therapy and reduce dropout in conservative treatment [5-8]. In fact, through the application of scoliosis personality questionnaires, interesting results have been found.

In this sense, Rivett et al. [9] have demonstrated the existence of significant differences with reference to personality in relation to the adherence to conservative treatment: patients who adhered to treatment had a superior HRQL than the noncompliant ones and showed different psychological profiles.

Before any treatment, Misterska et al. [5] proved the absence of psychopathological traits in evaluated adolescent girls but a high level of self-criticism was appreciated.

A Korean study [6] obtained different results. When evaluating a population of 19-year-old boys with AIS, they found out that these patients presented a higher incidence of neurosis, schizophrenia and personality disorders compared to a control group.

With reference to the application of projective tests, two studies [10,11] reached similar results. Using Rorschach and Bender test, Bengtsson et al. [10] found a difference in psychosocial adjustment in women with scoliosis. Superficially, the subjects seemed well adapted but from further analysis, they presented hypersensitivity and insecurity, with a tendency to dysphoric mood. Similarly, an Italian study [11] valued a discrepancy between a conscious body image and a deeper aspect of feelings of inferiority and insecurity through several projective tests (Raven’s Progressive Matrice, Human Figure Drawing (HFD) and Sacks’ battery).

Referring to research on patients with AIS, there is a lack of studies linking HRQL with the personality of these patients. However, in other areas, the existence of a relationship between personality and HRQL has been shown, such as in pediatric cancer patients [12] or with unintentional injuries [13] or young adults with asthma [14].

Considering that the studies on the personality of AIS patients are limited as well as the research on the relationship between HRQL and personality traits, despite their potential importance to the treatment, it seems appropriate for the authors to deepen these areas in a sample of patients with AIS.

In the present research, the authors assessed the hypothesis that patients suffering from AIS presented an emotional experience influenced by their disease and that there was a relationship between the aspects of their personality and their HRQL.

## Material and methods

This is a descriptive, cross-sectional, qualitative and quantitative study.

As inclusion criteria, patients (adolescents and young adults) with AIS, aged between 10 and 30 years with requirements for physiotherapy treatment were chosen. The requirements for physical therapy were defined by RM: patients with progressive AIS treated with rigid brace treatment as part of the treatment protocol; patients with AIS in mature age, Risser > 3 and more than two post-menarche years, without brace, still unconfirmed stability or stable but symptomatic (SRS-pain or Self-image or Function values < 4).

The study was carried out in a private center specialized in the conservative treatment of scoliosis (Spain). The collection time of the sample lasted 4 years (01/07/2008-29/07/2011) during the summer intensive physiotherapy course.

The test was carried out as follows: on the first day of the course, an informed consent to participate to the study was signed by the patients and their families (if they were underage) while a demographic- clinical data questionnaire, HFD and SRS-22 were administered to the patients.

Procedures and information how to fill in the tests were explained by a doctor and a psychologist. The questionnaires and the HFD were executed in group under the supervision of four observers (one doctor, two physical therapists and a psychologist). The observers had the function to give explanation if required and to ensure the quality of testing (reducing the mutual influence between subjects). The tests were administered as follows: first, the demographic and clinical data were collected, then a blank paper and a pencil were provided for the test of HFD and finally the SRS-22 was given.

Later, two psychologists with experience in HFD evaluated 30 emotional indicators [15] for each drawing. Before scoring, as the two evaluators had noted a high incidence of omission of facial features and of the shading of shoulders, they made the decision to include in the evaluation two more indicators: 31. Omission of facial features and 32. Shading of shoulders. The evaluation of the 32 indicators was separately performed by the evaluator 1 (E1) and the evaluator 2 (E2). After the scoring, different types of non-dressed figure were observed. Therefore, it was decided to separate the original indicator “nude figure/genital” into two: “nude figure” and “genitals”. To reduce inter-observer variability, “nude figure” indicator was evaluated jointly.

### Instruments

The instruments used were a questionnaire of demographic and clinical data, the HFD and the SRS-22.

The demographic- clinical data questionnaire was developed ad hoc by the authors for this study. It includes questions about gender, age and prior treatments for scoliosis. It is a self-administered questionnaire.

The drawing of the human figure (HFD) is a projective test of personality in which the participant is asked to draw a person. Each participant was given a blank sheet of paper and a pencil. Rubber was not used. According to Machover [16], a drawn figure is closely related to the impulses, anxieties and conflicts of the person.

Thus, the authors considered that the HFD personality instrument, by its nature, could assess deeper aspects related to the emotional area and body image in patients with AIS. In addition, this instrument has been used complementarily with other instruments in populations with physical illnesses, as patients with juvenile idiopathic arthritis [17], women with premature ovarian failure [18], burnt children [19] or people with hearing deficits [20].

Despite the criticism for its questionable psychometric characteristics, the instrument is quite popular in the clinical practice [21]. Although the calculation of the reliability is not very clear and its research is quite old, studies show a high value to test- retest [22] while inter-observer reliability obtained values above 0.80 in different studies [21].

In the present research the study by Koppitz [23] was taken as reference. The author developed a list of 30 indicators for the analysis of the drawings, examining 1856 drawings of children between 5 and 12 years-old. The indicators were divided into three categories: 1. General (9 items): related to the quality of the drawing; 2. Specific (13 items): items not common in HFD; 3. Omission (8 items): corresponding to the lack of 8 expected body elements.

The general indicators are: 1. Poor integration; 2. Shading face; 3. Shading of body and/or limbs; 4. Shading of hands and/or neck; 5. Marked asymmetry of the limbs; 6. Slanting figure (tilt in 15° or more); 7. Tiny figure; 8. Big figure; 9. Transparencies.

The specific indicators are: 1. Tiny head; 2. Crossed eyes; 3. One or more teeth; 4. Short arms; 5. Long arms; 6. Arms clinging; 7. Big hands; 8. Handless; 9. Legs close together; 10. Naked figure/ Genitals; 11. Monster; 12 Several figures; 13 Clouds, rain, snow, flying birds.

The Omission indicators are: 1. No eyes; 2. No nose; 3. No mouth; 4. No body; 5. No arms; 6. No legs; 7. No feet; 8. No neck.

In addition, the authors added three more indicators. The 31st indicator “Omission of facial features ” assesses whether the subject draws only the outline of the face and does not draw any features inside. This indicator was introduced after observing its high incidence on the sample and would be related both in adolescents and in adults with a tendency toward superficiality, evasiveness [24], or isolation [25]. According to Hammer [26], the omission of significant details is related to the use of defenses such as avoidance and feelings of emptiness.

The 32nd indicator, “Shading of shoulders”, assesses the presence of shading with reference to the shoulder area. Shading is always related to anxiety [16,26]. Thus, the shading on the shoulders refers to anxiety associated with this part of body.

**Figure 1:**
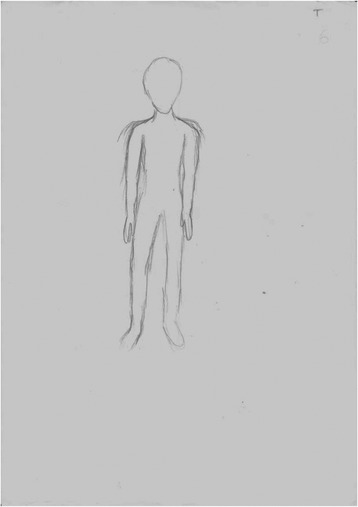
**Silhouette.** Example of the *Silhouette* category.

Finally, the original category “Naked figure/genitals” was split into two (“Naked figure” and “Genitals”). Concerning “Naked figure”, three possibilities were detected: 1. “Naked” (a person without clothes, including the drawings of genitals or other such as navel), 2. “Dressed” (clothes are drawn); 3. “Silhouette” (only the outline of the person is drawn: no other features in the body; the person is neither naked nor dressed) [Figure 1].

The SRS-22 is a HRQL questionnaire expressly constructed for patient with scoliosis by Asher et al. [3]. It consists of 22 items pertaining to five dimensions: 1. Function/activity (5 items), 2. Pain (5 items), 3. Self-image (5 items), 4. Mental health (5 items) and 5. Satisfaction with treatment (2 items). In this study, the authors did not consider this latter domain because the sample consisted of patients with diverse types and at different stages of treatment.

Each item is scored in a 5-point Likert scale with higher score indicating better HRQL. The score for each domain and the subtotal (that is the sum of the four domains regardless of the Satisfaction one) ranges from a minimum of 1 to a maximum of 5 points.

The questionnaire has good psychometric properties: internal consistency (Cronbach α = 0.86), reliability (r = 0.9) and concurrent validity (r = 0.7) [3].

### Statistical analysis

Descriptive analysis for the demographic and clinical characteristics of the sample was performed with regard to age, gender and prior treatment; SRS-22 descriptive scores were assessed, too. In addition, for each emotional indicator, their frequencies evaluated by the evaluators (E1, E2) and kappa index (k) to estimate the inter-observer agreement were performed.

With reference to the inter-observer concordance, a value of k ≥ 0.6 was considered, estimating as satisfactory k = 0.61- 0.80 and very satisfactory k = 0.81-1.00 [27].

The first hypothesis tests a relationship between HRQL and the emotional indicators. The authors expected worse HRQL to be related to a higher number of indicators. To explore it, the authors performed a Student’s t test for independent samples (depending on whether the indicator, dichotomous variable, was present or not); in the case of the Naked figure variable, an analysis of variance (ANOVA) was performed for one factor, as the main variable had three levels.

The second hypothesis to be tested was about the correlation between age and those emotional indicators associated to anxiety. Specifically, it was expected that older patients present more anxiety problems (shading indicators in the HFD) as they may be more aware of their illness. To study the relationship between age and emotional indicators, a chi-squared test was performed, as the variables were nominal and dichotomous. For this aim, the authors divided the sample into two groups: 1) the patients aged between 9 and 14; 2) the ones aged between 15 and 26. In addition, the authors made the hypothesis that age would negatively correlates with the SRS-22 scale of Mental Health: older patients presented more emotional problems. This correlation was studied through Pearson coefficient as they are quantitative variables.

With regard to the third hypothesis, a relationship between brace treatment and indicators related to negative emotions was expected, as a brace treatment is considered stressful. For this hypothesis, a Chi squared statistical test was carried out with the emotional indicators and the treatment group (braced/not braced). Furthermore, the hypothesis about a difference in HRQL between the group treated with a brace and the untreated one was raised. This hypothesis was tested by Student’s t test for independent samples.

Statistical software SPSS 18.0 was used. Statistical significance was set at p <0.05 was.

## Results

The sample consisted of 50 patients; 92% (n = 46) were women. 12 patients were not treated, while the rest (38) were under conservative treatment: 26 of the patients were doing physiotherapy while 27 were under brace treatment (13 of them with full time modality).

The patients were in the 9–26 age range (mean age = 16). The age distribution can be seen in Table 1. The age of one subject was unknown.

**Table 1:** **Frequency distribution for age**

**Age**	**Frequency**
9	1
11	5
12	2
13	4
14	10
15	5
16	8
17	4
19	1
21	1
22	3
24	2
26	3
Total	49

At the time of the study, 54% (n = 27) wore a brace in the following way: 26% (n = 13) full-time (n = 23 hours) and 28% (n = 14) part-time (10 hours); the remaining 46% of the sample did not wear a brace. 52% (26) had realized some kind of physical therapy in the past.

The mean scores for the SRS-22 questionnaire were: Function = 4.1 (SD = 0.5); Pain = 4.3 (SD = 0.7); Self Image = 3.2 (SD = 0.7) and Mental Health = 4 (SD = 0.6); Subtotal = 3.9 (SD = 0.4).

The HFD categories with inter-observer concordance values considered good or very good (index k ≥ 0.6) were 12. In Table 2, k values and the frequencies of the categories according to the two evaluators (E1 and E2) are reported. Referring to five categories, k coefficient value was zero since the category was recorded as absent for both observers (“No body” “Clouds, rain, snow, etc.”, and “Several figures”) or only for one of them (“Tiny head” or “Genitals”).

**Table 2:** **Values of k coefficient and frequencies according to the two evaluators (E1, E2)**

**Categoría**	**k**	**E1 Frecuency**	**E2 Frecuency**
1. Poor integration	0.2	22% (11)	26% (13)
2. Shading face	0.3	22% (11)	44% (22)
3. Shading of body and/or limbs	0.3	58% (29)	72% (36)
4. Shading of hands and/or neck	0.6*	34% (17)	56% (28)
5. Marked asymmetry of the limbs	0.3	12% (6)	32% (16)
6. Slanting figure	0.8*	8% (4)	12% (6)
7. Tiny figure	0.4	20% (10)	48% (24)
8. Big figure	0.2	14% (7)	2% (1)
9. Transparencies	0.1	16% (8)	6% (3)
10. Tiny head	-	6% (3)	0
11 Crossed eyes	0.2	18% (9)	8% (4)
12. One or more teeth	1*	2% (1)	2% (1)
13. Short arms	0.3	8% (4)	14% (7)
14. Long arms	−0.3	2% (1)	6% (3)
15. Arms clinging	0.5	26% (13)	14% (7)
16. Big hands	−0.3	6% (3)	2% (1)
17. Handless	0.5*	22% (11)	12% (6)
18. Legs close together	0.7*	16% (8)	10% (5)
19. Genitals	-	6% (3)	0
20. Monster	1*	2% (1)	2% (1)
21. Several figures	**-**	0	0
22. Clouds, rain, snow, flying birds	-	0	0
23. No eyes	0.3	34% (17)	18% (9)
24. No nose	0.4	40% (20)	26% (13)
25. No mouth	0.3	34% (17)	18% (9)
26. No body	**-**	0	0
27. No arms	1*	1 (2%)	1 (2%)
28. No legs	1*	1 (2%)	1 (2%)
29. No feet	0.9*	10% (5)	8% (4)
30. No neck	1*	2% (1)	2% (1)
31. Omission of the features of the face	0.7*	36% (18)	34% (17)
32. Shading shoulders	0.6*	48% (24)	52% (26)

*k ≥ 0.6.

Table 3 shows the frequencies of the 2 sub categories of the indicator “Naked figure”. 58% of the “dressed” indicator was observed against the 42% of the “not dressed” ones.

**Table 3:** **Frequency and percentage of the “Naked figure” category**

**Category**		**Frequency**	**Percentage**
Dressed		29	58%
Not dressed	Silhouette	10	20%
	Naked	11	22%

Following the descriptive analysis of the added indicators, a high number of subjects shadowing the shoulders was found for a value almost equal to half of the sample (E1 = 48%; E2 = 52%).

Furthermore, calculating the total frequency of the 33 emotional indicators for subjects, the average of indicators per person was between 22.74 (E1) and 22.70 (E2), (range of values: minimum = 17- maximum = 27).

The first hypothesis regarding the relationship between HRQL and personality was not confirmed since no significant values were found in any of the tests performed.

With reference to the second hypothesis, the first age group (9 to 14 years) consisted of 22 subjects; the second group (15–26 years) consisted of 27. The chi-squared test revealed that only the category “Shading of body and limbs” presented a significant dependence on age: the older group drew more this indicator, as it can be seen in Table 4.

**Table 4:** **Contingency table “Shading Body” × age**

**Emotional indicators**	**Age**		**Chi Squared value**	**Significance (bilateral)**
	**9-14**	**15-26**		
Shading of body and/or limbs (E1)	6	23	16.8	0.001
Shading of body and/or limbs (E2)	13	23	4.2	0.04

Chi-Squared Test.

Moreover, a significant correlation was found between Mental health and age. Although it was low (r = −0.3; p = 0.04), the hypothesis with reference to age was accepted: the older the patients, the more levels of tensions and poorer mental health they presented.

The last hypothesis on the impact of treatment on personality was rejected as no significant chi-squared values were found between the two treatment groups and the emotional indicators. On the other hand, there were no significant differences between the two groups (braced / no braced) with reference to the results of SRS-22Mental Health dimension.

## Discussion

In this study the authors intended to investigate the most relevant aspects of personality and HRQL in young patients with AIS. To study the emotions, the HFD projective technique has been used since this test is considered to be related to the impulses, anxieties and conflicts of the drawing person [16]. As the measurement process always involves some degree of error, the authors have chosen to reduce it through a double assessment (inter-observer concordance) of emotional indicators (only one indicator has been assessed jointly).

To study the HRQL, the SRS-22 questionnaire specific for people with scoliosis was used. The mean value of Subtotal found in this survey was 3.9 (range: minimum Auto Image = 3.2; Pain maximum value = 4.3); however, the authors believe that the values of the questionnaire could be enriched with data from HFD. Thus, this tool was included in the design and procedures of the study.

In this sense, the amount of emotional indicators observed in each drawing suggested the presence of some difficulties in patients with AIS. Among all the indicators, the presence of shading shoulders and shading hands/neck was detected. Shading is a general indicator of anxiety: it expresses concerns or tensions associated with the shaded part of the body [16,26]. Shading shoulder was present in almost half of the subjects (48% - 52%) and this datum was of great relevance because population with scoliosis often presents asymmetric shoulders and muscular tension. The shading of the shoulders would be associated with emotional stress related to the shoulders, which may be linked to body image or other type of tension that should be explored. The shading of hands and neck (34% -56%) can express a more symbolic kind of tension. Machover [16] considers hands a contact element and shading could manifest a “tension” in the interaction with the environment.

In addition, the omission of facial features (no eyes, no nose, no mouth) present in 34-36% of subjects seemed relevant, too. According to Hammer [26], omissions in general are referred to the use of defenses such as flight and a feeling of emptiness. Facial features omitted in adolescents and adults have a value of evasiveness and superficiality [24] and can also express a possible tendency toward isolation [25]. Also, the complete omission of features throughout the body (“silhouette”) present in 20% of the drawings could be interpreted as a total isolation and evasiveness. The silhouette evokes a “sexless ghost”, being only an outline of an empty interior.

Besides analyzing the category “naked figure” (modified by the authors), it was observed that 22% of subjects did not draw clothes. The presence of sexual organs is considered rare, except in the case of artists or people with severe pathology [16]. However, genitals also appear in the drawings of adolescents [28] reflecting pubertal changes, as it is the case of the patients of this study.

Besides, almost all the subjects (98%) did not draw teeth. Since teeth are associated with aggressive or sadistic tendencies [16,26], one could argue that the studied patients had a low expression of aggressiveness. This result could confirm the Polish study [29] conducted in a sample of patients a month before surgery, where the percentage of inner rage (not expressed, inhibited or hidden) was higher than the level of anger expressed.

With reference to the above hypotheses, the correlation between HRQL and the personality aspects of patients with AIS could not be verified. This result was contrary to what it was expected. Probably this could be explained by a possible discrepancy between conscious aspects, emerging from the SRS-22 questionnaire, and deeper aspects, detected with HFD.

Instead the second hypothesis concerning a relationship between age and emotion was confirmed: getting older, the frequency of the shading body and limbs increases and Mental Health worsens (although in the latter case the correlation coefficient was very low). The shading of the body and limbs expresses a general concern or a physical strain about the level of the body. In general, body tension can be explained as a physical dimension or as a symbolic dimension related to concerns and fears about the body in general [30].

This second hypothesis was raised from the observation that younger patients seemed little aware of the problem as scoliosis is often not painful and has a silent manifestation; however with the passing of the years, through several visits, X-rays, treatments, etc., the person might become more aware of the problem and express more concerns. On the contrary, the third hypothesis concerning the relationship between treatment and personality or between treatment and Mental Health could not be accepted. This result did not confirm our hypothesis as we considered that patients treated with brace should have experienced more negative emotions or worse mental health compared to the untreated group. Moreover, in this study, the brace treatment appeared to have no impact on the personality of patients. Possibly a further study would have to consider variables as the last of treatment, time modality, kinds of brace, or other factors specifically related to the treatment.

Our research has different limitations. As a projective test, HFD instrument has not the robustness of a quantitative tool. For this reason, two raters scored it. However, only 12 of the 32 indicators had a k value ≥0.6. Furthermore, the lack of a control group made hard to compare the results with those of a healthy population. Besides, in further studies, it would be interesting to use a quantitative personality test and to compare it with SRS-22 outcomes.

## Conclusions

In the studied sample of adolescents and young adults with AIS, mean values of HRQL were medium- high. In half of the patients (48% -52%), the test of the human figure revealed difficulties related to physical and/or emotional stress in the shoulders. Moreover, a third of the sample (34% -56%) experienced a difficulty at a relational level. Similarly, 34-36% displayed a certain tendency to isolation or shyness while 20% showed a defensive style of avoidance. Finally, the majority of the sample (90%) indicated no aggressive tendencies.

A relationship between HROL, measured with the SRS-22, and the aspects of personality explored through the HFD was not confirmed. Age was directly related to worries about the body and to a worsened mental health. A negative impact of the brace on personality or on mental health was not confirmed.

The authors think that a psychological work aiming at reducing tension, promoting greater body awareness and developing relational skills could be a valuable aid to these patients, above all for adolescent ones, in order to prevent possible future problems.
